# Bistability and Oscillations in Gene Regulation Mediated by Small Noncoding RNAs

**DOI:** 10.1371/journal.pone.0017029

**Published:** 2011-03-17

**Authors:** Dengyu Liu, Xiao Chang, Zengrong Liu, Luonan Chen, Ruiqi Wang

**Affiliations:** 1 Institute of Systems Biology, Shanghai University, Shanghai, China; 2 College of Physics and Mathematics, Jinggangshan University, Ji'an, China; 3 Key Laboratory of Systems Biology, SIBS-Novo Nordisk Translational Research Centre for PreDiabetes, Shanghai Institutes for Biological Sciences, Chinese Academy of Sciences, Shanghai, China; Tel Aviv University, Israel

## Abstract

The interplay of small noncoding RNAs (sRNAs), mRNAs, and proteins has been shown to play crucial roles in almost all cellular processes. As key post-transcriptional regulators of gene expression, the mechanisms and roles of sRNAs in various cellular processes still need to be fully understood. When participating in cellular processes, sRNAs mainly mediate mRNA degradation or translational repression. Here, we show how the dynamics of two minimal architectures is drastically affected by these two mechanisms. A comparison is also given to reveal the implication of the fundamental differences. This study may help us to analyze complex networks assembled by simple modules more easily. A better knowledge of the sRNA-mediated motifs is also of interest for bio-engineering and artificial control.

## Introduction

Small noncoding RNAs (sRNAs) have been demonstrated in recent years to play crucial roles both in prokaryotes and eukaryotes [1]. These sRNAs may control diverse processes, including cell growth, death, development, and differentiation, by determining how and when genes turn on and off [2]–[5]. The regulatory roles of sRNAs have been a subject of research for the last several years, both experimentally and theoretically [6]–[10]. Although some of the RNAs have been well studied, the information about possible functions and biological significance of sRNAs still need to be fully understood due to the diversity of mechanisms by which sRNAs may regulate biological processes.

The mechanisms by which sRNAs exert their effects are diverse. It has been demonstrated that sRNAs play transcriptional regulatory roles only in a small number of cases. Like regulatory proteins, sRNAs can regulate expression of multiple target genes, and are themselves regulated by one or more transcriptional factors. Besides binding to the 5′ untraslated region of a target mRNA with specificity achieved through base-paring between the two RNA molecules, sRNAs can also significantly down-regulate target protein levels, yet do not notably affect their target mRNA stability [11]. In addition, sRNAs can also positively regulate protein expression by promoting ribosome binding to their target mRNAs [12], [13]. This leads to a natural question: what are the fundamental differences between different regulatory scenarios?

To address this question, we consider the minimal architectures with only one sRNA and one mRNA, which is a recurrent network motif mediated by sRNAs [10]. For instance, the module E2F1/miR-17 in the E2F1/miR-17–20/c-Myc network in human belongs to the scope of such a motif, in which E2F1 activates transcription of the miR-17 sRNA cluster and miR-17 mediates a negative feedback to E2F1 [4], [10]. Another example is the module RpoE/*rhyB*, in which RpoE activates transcription of the sRNA gene *rhyB* and *rhyB* in turn represses RpoE synthesis [9]. To investigate the sRNA regulation by binding directly to target mRNAs or to proteins, we construct mathematical models and compare the distinct features associated with the two scenarios. The study of the minimal architectures mediated by sRNAs may help us to analyze complex networks assembled by these modules more easily. A better knowledge of the sRNA-mediated motifs is also of interest for the bio-engineering or artificial control of specified components, interactions, and even network functions.

Nonlinear phenomena in cellular networks such as bistability and oscillations have been intensively investigated primarily across the coding region of genes, producing mRNAs for translation. However, this view has been turned recently, especially more and more experimental evidences showed that sRNAs can regulate a broad range of biological processes [2]–[5], [14]. For example, efficient degradation and prioritization of targets mediated by sRNAs have been investigated by building simple models of two simple motifs involving sRNAs [15]. Therefore, it is getting necessary to take different regulation scenarios mediated by sRNAs into account and study nonlinear phenomena induced by them, especially bistable and oscillatory phenomena, which are very common in cellular systems. The complex phenomena revealed by the sRNA regulation can help us to understand the crucial roles of sRNAs in gene regulation and further physiological functions.

In this work, we focus mainly on understanding how bistability and oscillations are induced by the interplay between two RNAs and one protein. Based on the different regulatory scenarios mediated by sRNAs, two mathematical models are constructed and quantitative comparison between them is given. Both scenarios largely differ in the onset of bistablity and oscillations. It is hoped that the difference will generate a detailed and precise insight of sRNA-mediated regulation.

## Results

**Figure 1:**
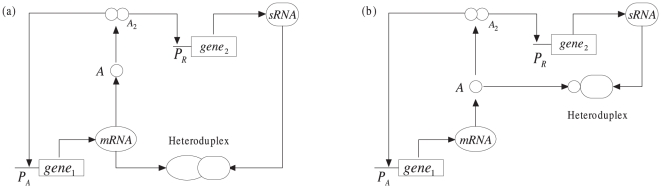
Schematic description of the two scenarios. (a) Scenario I: post-transcriptional regulation by binding of a sRNA and an mRNA. (b) Scenario II: translational repression by binding of a sRNA and a protein causes inactivation of the protein.

We introduce two scenarios with sRNA operating differently to describe the interplay of an mRNA 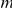, a sRNA 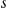, and a protein 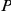, as shown in Fig. 1. Although the detailed mechanism of post-transcriptional regulation by sRNAs is not fully understood, evidence for the functional roles of sRNAs is accumulating. One of the sRNA regulations is that it directly affects levels of its target transcripts by accelerating their degradation rates and therefore lower their expression levels. This is achieved through binding of sRNAs by partial nucleotide sequence complementarity to their target mRNA sequences. Once an mRNA reaches the complex mRNA-sRNA, it is not available for translation, causing reduction in the encoded protein level, as shown in Fig. 1(a).

In the scenario I, the encoded protein homodimer activates transcription of the mRNA and sRNA. To mathematically describe the dynamics of such a post-transcriptional regulation by a set of differential equations, we outline the reactions consisting of the mRNA 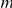, sRNA 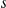, and protein 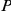 as follows 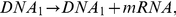
(1)

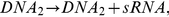
(2)

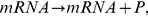
(3)

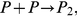
(4)

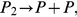
(5)

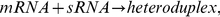
(6)

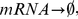
(7)

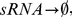
(8)

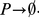
(9)Note that we just consider the heteroduplex association here. When the dissociation is considered, the heteroduplex can release the mRNA and sRNA and the heteroduplex concentration needs to be treated as a dynamical variable. Of course, when the association-dissociation reactions are assumed to be faster than other reactions, the heteroduplex variable can be eliminated [16].

In the scenario I, the binding of the mRNA and sRNA by basepairing forms the heteroduplex and enhances the degradation of the mRNA. Using standard quasi-steady-state approximation, we can obtain the simplified model as follows 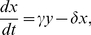
(10)

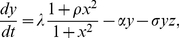
(11)

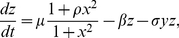
(12)where 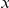, 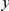, and 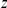 are the concentration of the protein, mRNA, and sRNA, respectively, 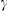 is the synthesis rate of the protein, 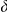, 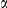, and 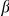 are the degradation rate of protein, mRNA, and sRNA, respectively, 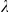 and 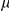 are the effects of the transcription factor on each gene, respectively, 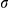 is the association rate of the two RNAs, and 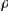 is the increase of the transcription rate due to the binding of the activator to the promoters. In this model, besides the linear degradation of the mRNA and sRNA, the nonlinear degradation of mRNA and sRNA is induced by the association of two RNAs.

In the scenario I, the regulation is mediated by the mRNA-sRNA interaction, in which the sRNA acts as a fine-tuner of gene regulation through binding of the sRNA by partial nucleotide sequence complementarity to its target mRNA sequences. Besides this scenario, sRNAs can also significantly down-regulate target protein levels, yet do not notably affect their target mRNA stability by blocking translation initiation or post-initiation steps or other significantly different mechanisms [11], [17], [18]. Despite the differences between various mechanisms, sRNAs significantly down-regulate their target protein levels. However, until recently, most studies on sRNA-mediated regulation focus on the principles of sRNA-mRNA interaction, while studies on translational repression are few because the mechanisms of translational repression remain a matter of debate. Pioneering studies have shown that sRNAs can bind to proteins. For example, AC4, a unique virus-encoded post-transcriptional gene-silencing suppressor protein, binds to and presumably inactivates mature sRNAs and thus blocks the normal sRNA-mediated regulation of target mRNAs [19]. Actually, many noncoding RNAs can directly bind to proteins. For instance, the bacterial 6S RNA and the eukaryotic B2 RNA directly target RNA polymerases, while the 7SK and steroid receptor RNA activator (SRA) bind to and regulate the activity of transcriptional factors [20]. In some sense, similar to the formation of the hetorpduplex by binding of the mRNA and sRNA, in which the mRNA can not be translated into a protein, the binding of sRNAs and proteins also inactivates the proteins, e.g., transcriptional factors or enzymes. Similar to the sRNA-induced mRNA cleavage [21], we just use a relatively simple manner to model the translational repression as follows, as shown in Fig. 1(b).
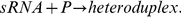
(13)


It is obvious that the sRNA can play an important role in repressing the protein in the scenario II. When replacing [6] by [13], the full rate equations can be similarly obtained as follows 
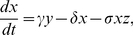
(14)

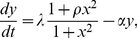
(15)

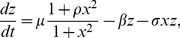
(16)where 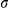 denotes the association rate of the sRNA and protein. In other words, the sRNA and protein co-degrade nonlinearly at a rate 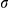 besides their respective linear degradation. The difference between the two models lie in the formation of different complexes, i.e., the mRNA-sRNA complex in the first scenario and sRNA-protein in the second scenario. Since the transcription is regulated by the homodimer, the Hill coefficient in (11)–(12) and (15)–(16) is 2.

To investigate the dynamical potential of sRNAs in the regulation of gene expression either by binding directly to target mRNAs or proteins, we will next examine and compare the two models with computational analysis. To determine fundamental differences in the two scenarios, it would be of interest to investigate similar parameter values in the two models. Here, bistable and oscillatory phenomena revealed by the sRNA regulation show that sRNAs may play crutial roles in gene regulation and further physiological functions.

### Bistability induced by the sRNA

Bistability, i.e. the capacity to achieve two alternative internal states in response to different stimuli, exists ubiquitously in cellular systems. It is a defining characteristic of a switch. Cells can switch between two internal states to accommodate environmental and intercellular conditions. It is increasingly becoming clear that such two or multiple discrete and alternative stable states are generated by regulatory interactions among cellular components. It has been found in both synthetic and natural biomolecular networks, including gene regulatory networks [23], signal transduction networks [24], [25], and metabolic networks [26]. Bistability has fundamental biological significance, notably in cell differentiation [27], [28], cell fate decision [29], adaptive response to environmental changes [30], regulation of cell-cycle oscillations during mitosis [31], etc. However, all bistability is produced primarily across the coding region of genes. Recently, switches involving noncoding sRNAs have been studied experimentally [2], [3] or theoretically [32]. Similarly, sRNA-mediated cell fate decision has also been extensively investigated [4], [5].

**Figure 2:**
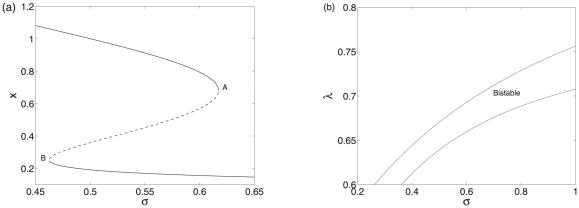
Bifurcation diagrams of model I. (a) The bifurcation diagram with 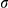 as a control parameter. (b) The codimension two bifurcation diagram with 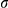 and 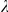 as control parameters. Other parameter values are 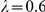, 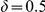, 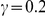, 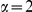, 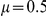, 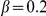, and 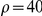.

To highlight distinct features associated with the two scenarios, we consider similar parameters for both implementations. When analyzing the two models with computational analysis, we choose the rate constant 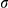 as a governing parameter because it is the key parameter responsible for the degradation mediated by the sRNA. The bifurcation diagram of the system (10)–(12) is shown in Fig. 2(a). It can be seen that bistability occurs only for intermediate association rate, where A and B are the saddle-node bifurcation points. The two stable equilibrium loci monotonically decrease with 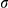 because the formation of the sRNA-mRNA complex irreversibly inactivates both RNAs and further down-regulate the protein levels. In other words, the sRNA base pairs with the garget mRNA at a rate 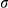 which accounts for the probability for the sRNA co-degraded with the mRNA. The base pairing blocks the bindings of the ribosome to the mRNA and thus negatively regulates the translation process. Therefore, increasing the co-degradation rate will induce the decrease of both RNAs and further the protein.

Different from the case of monostability, where a higher degradation rate corresponds to a lower protein concentration and a lower degradation rate corresponds to a higher protein concentration due to the negative regulation mediated by the sRNA, when the association rate lies between the two saddle-node bifurcation points, despite the negative regulation mediated by the sRNA, a higher degradation rate may correspond to a higher protein concentration or a lower degradation rate may correspond to a lower protein concentration, depending on the initial conditions. In such a case, the convergence depends on not only the interplay of the sRNA and mRNA but also their initial conditions. Moreover, cellular processes at the molecular level are inherently stochastic. The origin of stochasticity can be attributed to random transitions among discrete biochemical states, which are the source of inherent fluctuations. There can be two sources of noise. First, the inherent stochasticity in biochemical processes such as binding, transcription, and translation generates the intrinsic noise due to random encountering, whose relative magnitude is proportional to the inverse of the system size. Second, variations in the amounts or states of cellular components due to discrete numbers or the external environment generate the extrinsic noise. Such noises are believed to play especially important roles when species are present at low copy numbers. Such kinds of noise may induce switching between the two stable states. When the switching occurs, a higher association rate may correspond to a higher protein concentration or a lower degradation rate may correspond to a lower protein concentration.

In order to investigate the interplay between the transcription factor 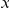 and the sRNA, we consider the effects of variations in parameters 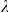 and 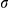 on the dynamics of model I. The codimension two bifurcation diagram is shown in Fig. 2(b). It can be seen that the bistability region varies with the two control parameters. When there is no the negative post-transcriptional regulation, i.e. at 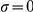, the system is monostable. Therefore, the negative regulation mediated by the sRNA can induce bistability. With increasing of 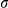, bistability occurs, depending on the value of the parameter 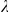.

### Oscillation induced by the sRNA

Besides the bistability, the sRNA can also induce some non-steady-state behavior, e.g. the sensitivity and large-amplitude oscillations induced by the sRNA-17–92 cluster [4]. It was also shown that the effects of sRNAs on gene expression can be destabilizing, i.e. promote the occurrence of oscillatory expression dynamics [33]. Also, sRNAs are always related to circadian clock, e.g. oscillations in four distinct Arabidopsis sRNAs, miR-167, miR-168, miR-171, and miR-398 appear to be a response to light and are not governed by the circadian clock [34]. Researchers sussed out that recruited motifs mediated by sRNAs enhance the fidelity, robustness and flexibility in temporal regulation [10]. Although recent work has implicated roles of sRNAs in development and in disease, the expression and function of sRNAs in gene expression still need to be extensively characterized.

**Figure 3:**
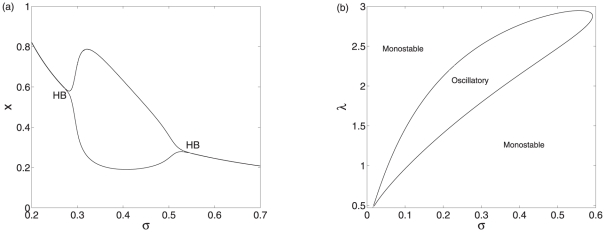
Bifurcation diagrams of model II. (a) The bifurcation diagram of model II with 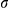 as a control parameter. (b) The codimension two bifurcation diagram of model II with 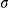 and 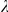 as control parameters. Other parameter values are 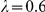, 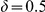, 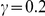, 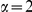, 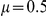, 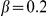, and 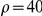.

In the model I, bistability occurs as the degradation rate 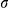 increases monotonically. In the model II, when the system is stable, concentration of the protein still decreases with 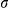 because a larger association rate means lower protein synthesis. The stability of the equilibria can be changed with the variation in 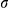. At a smaller 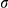, the unique equilibrium is stable. As 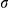 increases gradually, the stability of the equilibrium transforms from stable to unstable, meanwhile sustained oscillations appear. When the parameter 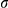 keeps on going out of the range of oscillations, oscillations vanish and the unique equilibrium regain its stability. There exist two supercritical Hopf bifurcations as the degradation rate 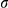 increases, as shown in Fig. 3(a). The first one is supercritical, resulting in a stable branch of limit cycles. While the second one is also supercritical because the equilibrium branch loses the stability going left and the periodic orbit branch goes left too. Therefore, besides the bistability, the negative regulation mediated by the sRNA can also induce oscillations.

To investigate the interplay between the transcription factor and the sRNA, we consider the effects of variations in the parameters 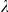 and 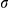 on the system dynamics. The dynamics of the model II can be oscillatory or monostable, depending on the parameter values of 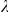 and 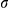, as shown in Fig. 3(b). Therefore, the dynamics can be significanly affected by the regulation mediated by the sRNA. When the co-degradation rate is large enough, the system will converge to a stable equilibrium and no oscillations can occur. Even at a intermediate 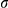 value, the occurrence of oscillations also depends on the value of 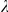. Therefore, the oscillations are induced by the interplay of two RNAs and the transcription factor. Such an oscillator mediated by the sRNA belongs to the scope of oscillators by amplified negative feedback loops [35].

The bistability in the model I and oscillations in the model II have been discussed to show that complex dynamical phenomena can be induced by the negative regulation mediated by the sRNA even in the simple models. Actually, bistability and oscillations can occur in both of the models. Next, we will compare the two models with computational analysis to investigate the fundamental differences between them.

### The comparison between the two scenarios

For both of the scenarios, we can compare the kinetics of gene regulation mediated by the mRNA-sRNA and sRNA-protein interaction. In order to give prominence to the distinct features associated with the two scenarios, we consider similar biochemical parameters for both of the implementations. Although it remains unknown what conditions are necessary for the occurrence of bistability or oscillation in the two models, the bifurcation diagrams of both models are useful to gain insight into different regulatory mechanisms. When bifurcation is performed, the most apparent property distinguishing both scenarios is the difference in the regions where bistability and oscillations occur.

**Figure 4:**
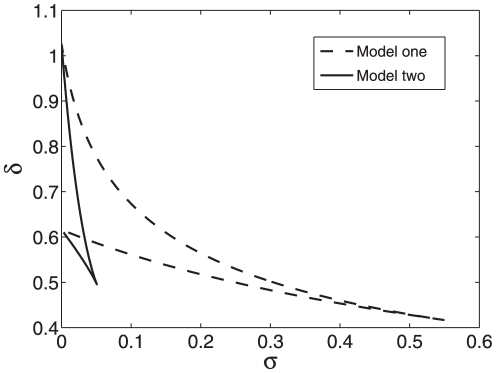
Bifurcation diagrams of the two models with 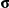 and 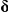 as control parameters. The regions enclosed by dashed and solid lines are the bistable regions of the two models. Other parameter values are 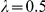, 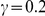, 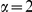, 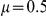, 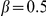, and 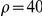.

The bifurcation diagrams of the two models with 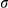 and 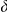 as control parameters are shown in Fig. 4. When there is no the regulation mediated by the sRNA, i.e. at 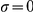, both systems are identical and the dynamics can be monostable or bistable, depending on the value of 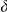. However, the scenario II, i.e. the translation repression, becomes monostable earlier when 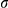 increases gradually. Therefore, the co-degradation mediated by mRNA-sRNA complex is more efficiently to induce bistability than the translation repression process. In other words, the post-transcriptional repression is more efficiently to produce bistability than the translation repression. When the association rate is large, only the co-degradation mediated by mRNA-sRNA can produce bistability. Actually, we can see that both scenarios can produce bistability at 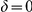 and intermediate values of 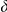. In other words, only intermediate protein levels can produce bistability. Due to a much faster degradation in the protein levels induced by the translation repression, the scenario II becomes monostable earlier than the scenario I. When the co-degradation rate 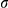 is large enough, both scenarios become monostable due to too low protein level.

**Figure 5:**
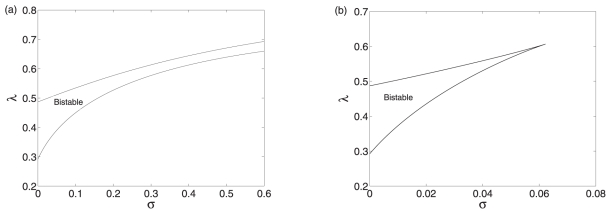
The bistability region of both models. (a) The bistability region of model I. (b) The bistability region of model II. The parameter values are 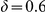, 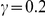, 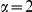, 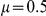, 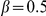, and 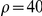.

We further check the effects of variation in parameters 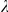 and 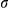 on both models when the value of 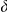 is located in the bistability region of Fig. 4. The bifurcation diagrams with 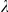 and 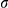 as control parameters are shown in Fig. 5. Both of the models are bistable for smaller 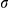 and intermediate 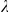. However for larger 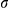, only the scenario II becomes monostable. The post-transcriptional regulation can be much easier to produce bistability than the translation repression regulation. Actually, for the scenario I, larger 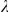 compensates the inefficiency in the protein levels needed to produce bistability and therefore the model I can still be bistable even at larger 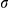. While for the scenario II, even larger 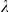 can still not compensate the inefficiency in the protein level needed to produce bistability due to much faster degradation in the protein level.

**Figure 6:**
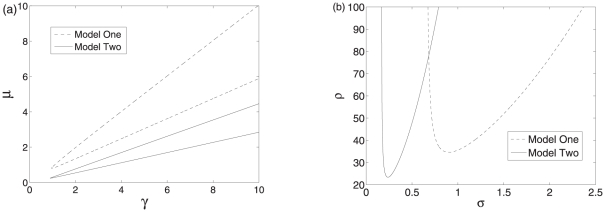
Bifurcation diagrams of the two systems. (a) The bifurcation diagrams with 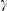 and 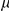 as control parameters. (b) The bifurcation diagrams with 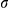 and 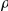 as control parameters. The regions enclosed by dashed and solid lines are the oscillatory regions of the two systems. Other parameter values are 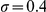, 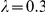, 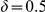, 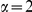, 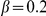, and 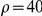.

To explore the effects of the production rate of the protein and the transcription factors on the dynamics of two systems, we take 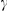 and 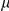 as control parameters. The codimension two bifurcations are shown in Fig. 6(a), where the regions enclosed by the solid and dashed lines are the oscillatory regions of the two systems. At smaller 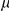 and 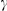, both of the two systems are not oscillatory. Therefore, large enough protein production and strong enough effects of the protein on sRNA are needed to produce oscillations. For a given larger 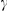, a larger 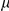 is necessary to induce oscillations for the regulation mediated by the mRNA-sRNA association. At the same time, the oscillatory region of the regulation mediated by the mRNA-sRNA association is also larger. Similarly, the codimension two bifurcation with 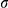 and 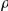 as control parameters are shown in Fig. 6(b). It can also be seen that the oscillatory region of the regulation mediated by the post-transcriptional repression is larger, indicating that post-transcriptional repression is more robust than translation repression in producing oscillations.

The analysis brought new insights into the possible roles for sRNAs, revealing that the interplay of mRNA, protein, and sRNA can play crucial roles in determining the cell fates. Especially, when bistability and oscillations are taken into account, the post-transcriptional repression is more efficient and robust than translation repression. The analysis points toward a diversity of mechanisms by which they may regulate transcription and translation so as to produce different functions such as bistability and oscillation.

### Interplay of nonlinear and linear degradation

The bistability and oscillations induced by the interplay of two RNAs and the protein have been analyzed. The analysis shows that the regulation mediated by the sRNA can produce both bistability and oscillations, depending on the parameter values. Especially, the post-transcriptional repression is more efficient and robust than translation repression in producing bistability and oscillations. Besides respective linear degradation in the RNAs and protein, there is also a nonlinear co-degradation mediated by the sRNA in the two scenarios. Such nonlinear degradation may play crucial roles in gene regulation. For example, it has been shown that nonlinear protein degradation is important for the robust operation as well as their evolvability in natural or engineered gene circuits [36]. We now turn to analyze the interplay of nonlinear and linear degradation in the sRNA. Similar analysis can be performed to the interplay of nonlinear and linear degradation in the mRNA and protein.

**Figure 7:**
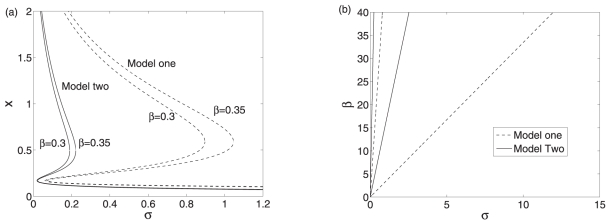
The bifurcation diagrams of the two models at 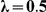. (a) The bifurcation diagrams with 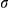 as the control parameter. (b) The bifurcation diagrams with 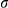 and 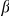 as control parameters. Other parameter values are the same as those used in Fig. 5.

We first consider the case in which both systems are bistable. The bifurcation diagrams are shown in Fig. 7. It can be seen that for both of the systems, larger 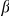 can produce much wider bistability region. In addition, different 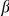 affects the low state more efficiently than the high one. Moreover, the bistability region of the scenario I is wider than that of the scenario II for the same 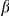, as shown in Fig. 7(a). The codimension two bifurcation diagrams with 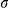 and 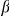 as control parameters are shown in Fig. 7(b). It shows that the scenario I, i.e. the post-transcriptional regulation mediated by the sRNA has larger bistability regime, meaning that the post-transcriptional regulation mediated by sRNA has a better robustness than the translation repression.

**Figure 8:**
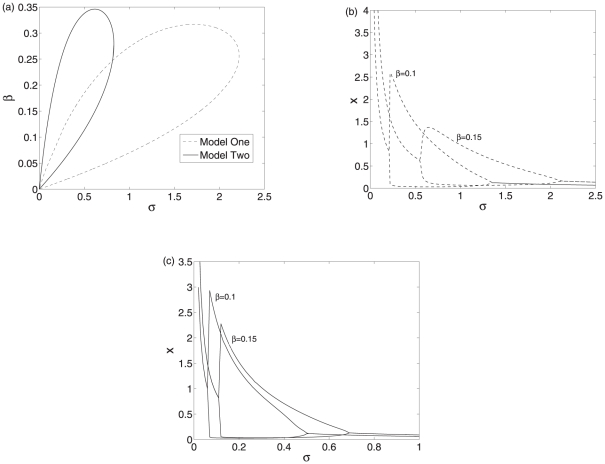
The bifurcation diagrams of the two models. (a) The oscillatory regions of the two systems. (b) The bifurcation diagrams of the first model. (c) The bifurcation diagrams of the second model. The parameter values are 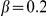, 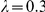, 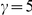, 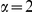, 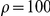, 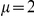, and 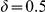.

To investigate the oscillatory case in the two scenarios, we still consider the relationship between 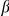 and 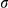. The bifurcation diagrams are shown in Fig. 8. It can be seen that both the linear and nonlinear degradation can play important roles in inducing oscillations. For both of the systems, the supercritical Hopf bifurcation values decrease with 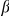. In other words, a smaller 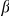 corresponds to a smaller supercritical Hopf bifurcation value of 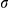. Moreover, the oscillatory regions of the scenario I is larger than that of the scenario II.

## Discussion

Gene regulation actually occurs essentially everywhere, including both coding and non-coding regions. The interplay of mRNAs, sRNAs, and proteins has been demonstrated to play crucial roles in gene regulation. In this paper, we have explored the dynamics of two minimal architechtures mediated by sRNAs. Especially, we introduced and compared two scenarios with sRNA operating differently, i.e. the post-transcriptional repression and translational repression. In both scenarios, there exist complex dynamics, e.g. bistablity and oscillation. The complex phenomena revealed by the sRNA regulation show that sRNA may play important roles in gene regulation and further physiological functions.

The key finding of our work is that different regulation mediated by sRNA has different features in inducing complex phenomena. In our simulations, we focused mainly on the bistability and oscillations induced either by binding of the sRNAs directly to target mRNAs or to proteins. We found that bistability and oscillations can only occur at intermediate association rates. Larger association rates may induce fast degradation in protein levels and the two scenarios inevitably converge to a unique stable state. In addition, as far as bistability and oscillations are concerned, the scenario I, i.e. the post-transcriptional regulation mediated by the sRNA, has a better efficiency and robustness than the translational repression. Moreover, the relatively slower degradation of protein induced by the post-transcriptional regulation can be compensated by stronger transcription activation so as to produce bistablity, while the inefficiency in producing bistability induced by much faster degradation of protein mediated by the translation repression can not be compensated. Besides the interplay of the two RNAs and protein, the interplay between the nonlinear and linear degradation may also play different roles in gene regulation.

Small non-coding RNAs are regulatory molecules that participate in diverse cellular processes. It has been shown that sRNA mediated feedback and feedforward loops are recurrent network motifs [10], e.g. the modules RpoE/*rhyB* [9] and E2F1/miR-17–20 [4], [10]. The study of these minimal architectures mediated by sRNAs may help us to analyze complex networks assembled by these modules more easily. A better knowledge of the sRNA-mediated motifs if also of interest for the bio-engineering or artificial control of specified components, interactions, and even network functions.

Besides the minimal architectures discussed in this paper, network motifs mediated by sRNAs with other architectures can be similarly analyzed. Actually, the mechanisms by which sRNAs exert their effects are diverse and until now only a few cases involving regulation by sRNAs were known. In addition to these general motifs mediated by sRNAs, many detailed regulatory processes involving sRNAs, e.g. the switch of FLO11 toggled by the sRNAs *PWR1* and *ICR1* [2] and modulation of circadian clock period and entrainment by miR-219 and miR-132 [14], need to be fully investigated. Further exploration of sRNA mediated regulation in the context of complex regulatory networks will provide a more comprehensive view on how gene expression is regulated at the systems level.

## Materials and Methods

In this work, two minimal architectures involving sRNA regulation are constructed. Especially, the effects of different regulatory mechanisms on bistability and oscillation are investigated. The dimer 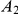 can bind to the promoters of genes 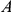 and 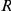 as their transcription factor, which can activate (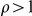) or repress (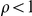) transcription of the two genes. The gene 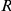 produces a kind of small noncoding RNA. Based on the two regulatory scenarios mentioned in the main text, the detailed common biochemical reactions can be described as follows: 
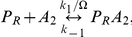


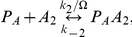


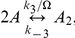


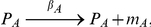


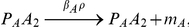


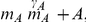


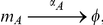





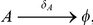


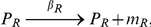


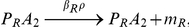


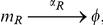
and the post-transcriptional repression and translational repression can be described by 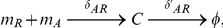
and
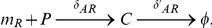
respectively.

We just show how to get differential equations from the above biochemical reactions for the scenario I, i.e. the post-transcriptional repression. The model of scenario II can be similarly obtained. These chemical reactions can be described by the following ordinary differential equations:
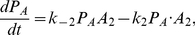
(17)

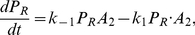
(18)

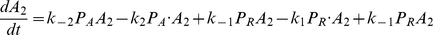


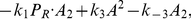
(19)

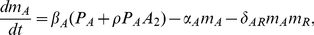
(20)

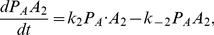
(21)

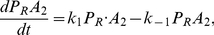
(22)

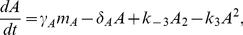
(23)

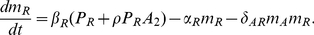
(24)


Assuming that the fast reactions are to be in their equilibria, i.e., by letting 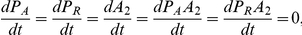
we can obtain the simplified system as follows 
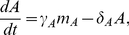
(25)

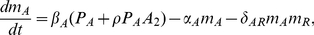
(26)

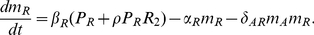
(27)


Introduce the association constants for DNA-binding and multimerization 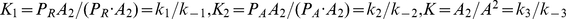. The conservation law is 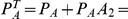
cons. and 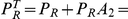
cons. Then, the simplified system can be rewritten as follows
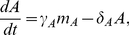
(28)

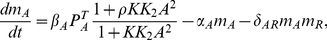
(29)

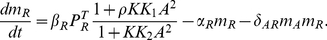
(30)Assuming that 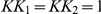 and letting 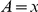, 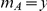, 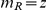, 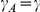, 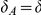, 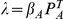, 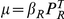, 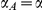, 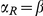, and 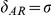, we can rewrite the above equations as follows 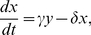
(31)

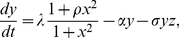
(32)

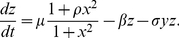
(33)

